# Growth charts for Thai children with Prader-Willi syndrome aged 0–18 years

**DOI:** 10.1186/s13023-020-01388-7

**Published:** 2020-05-06

**Authors:** Nantiya Mongkollarp, Thipwimol Tim-Aroon, Chusak Okascharoen, Khunton Wichajarn, Jeeraparn Phosuwattanakul, Nalinee Chongviriyaphan, Duangrurdee Wattanasirichaigoon

**Affiliations:** 1 Department of Pediatrics, Faculty of Medicine Ramathibodi Hospital, Mahidol University, Bangkok, Thailand; 2 Division of Medical Genetics, Department of Pediatrics, Faculty of Medicine Ramathibodi Hospital, Mahidol University, Bangkok, Thailand; 3 Division of Evidence-based Pediatrics, Department of Pediatrics, Faculty of Medicine Ramathibodi Hospital, Mahidol University, Bangkok, Thailand; 4 Division of Medical Genetics, Department of Pediatrics, Faculty of Medicine, Khon Kaen University, Khon Kaen, Thailand; 5 Division of Nutrition, Department of Pediatrics, Faculty of Medicine Ramathibodi Hospital, Mahidol University, Bangkok, Thailand

**Keywords:** Prader-Willi syndrome, Growth chart, Body height, Body weight, Head circumference, Southeast Asian

## Abstract

**Background:**

Prader-Willi syndrome (PWS) is a multisystem genetic disorder, which has a typical eating behavior and growth pattern. In the infancy period, children with PWS have low body weight followed by hyperphagia in later childhood. Disease-specific growth charts have been recommended for monitoring PWS patients. Previous literature demonstrated growth differences among individuals with PWS of different ethnicity.

**Methods:**

A retrospective multicenter study was performed in PWS patients from different areas of Thailand included collaboration with the Thai PWS support group during 2000–2017. Baseline characteristics and anthropometric data were reviewed. Both growth hormone and non-growth hormone received patients were included, but the data after receiving GH were excluded before curve construction. Growth charts for Thai PWS compared to the 50th normative centile were constructed using Generalized Least Squares (GLS) methods. Curve smoothing was performed by Fractional Polynomials and Exponential Transformation.

**Result:**

One hundred and thirteen patients with genetically confirmed PWS (55 males and 58 females) were enrolled. Fifty percent of patients were diagnosed less than 6 months of age. We developed growth charts for non-growth hormone treated Thai children with PWS aged between 0 and 18 years. A growth pattern was similar to other ethnicities while there were some differences. Mean birth weight of PWS patients was less than that of typical newborns. Mean adult height at 18 years of age in Thai children with PWS was lower than that in American children, but taller than Japanese. Mean weight of Thai PWS males at 18 years of age was more than those from other countries.

**Conclusion:**

This study is the first to document PWS-specific growth charts in Southeast Asian population. These growth charts will be useful in improving the quality of patient care and in evaluating the impact of growth hormone treatment in the future.

## Background

Prader-Willi Syndrome (PWS) is a multisystem genetic disorder presenting with infantile hypotonia, unique eating habits and growth patterns, short stature, typical facial dysmorphism, small hands and feet, intellectual disability, behavioral abnormalities, and hypogonadism. The prevalence of PWS is 1:10,000–1:30,000 live births, and it is considered the most common syndrome with morbid obesity [1].

Nutritional status in PWS can be classified into 5 phases [2, 3]: Phase 0 is defined as decrease of fetal movement and growth restriction in utero. Hypotonia and failure to thrive are noted in phase 1a during infancy period. During phase 1b (9-month to 2-year-old), patients can improve weight gain with normal growth velocity. At 2–4 years of age (phase 2a), children with PWS increase in weight gain without an increase in appetite. Phases 2b, between 4 and 8 years of age, patients increase their food-seeking behavior. Phase 3–4, children with PWS have hyperphagia and dramatically increased weight. Children with PWS typically do not have a growth spurt in puberty, which has been explained by hypogonadism [1]. For these reasons, growth charts for healthy children are not suitable for PWS patients. The American Academy of Pediatrics stated that PWS-specific growth charts should be used only for monitoring non-growth hormone treated patients [4]. Several previous reports have presented specific growth curves for PWS in the US, Germany, Korea, and Japan [5–9]. Although, the growth pattern of PWS are similar between different ethnicities, the actual height and weight of specific ethnic groups are somewhat different as noted in previous reports from East Asian, and from Caucasian populations [5–9]. Growth hormone (GH) treatment can lead to improved body composition, physical activity, and growth velocity in PWS children [6, 10, 11]. PWS patients with GH treatment would need disease-specific growth charts to evaluate response of the treatment.

In Southeast Asia, where the population size is approximately 640 million people, while it has not had its own PWS growth chart at the present time. Since Thai and Southeast Asian populations are ethnically closer as compared to Caucasian and East Asian groups, it is sensible to presume that the Thai PWS growth charts may be more applicable to those affected populations of Southeast Asian origin. Therefore, this study developed growth charts for Thai non-GH treated children with genetically confirmed diagnosis of PWS. The charts will be useful in improving growth monitoring and the quality of patient care as well as illustrating the importance of growth curve development in diverse populations.

## Methods

The study was a retrospective multicenter study during 2016–2018. Inclusion criteria were children with PWS diagnosed either by fluorescent in situ hybridization (FISH) for 15q11 using the Vysis probe’s protocol (Abbott Molecular) or *SNRPN* methylation specific PCR [12, 13]. The patients were recruited from the Thai PWS support group and three hospitals in different parts of Thailand including Ramathibodi Hospital in Bangkok, Maharat Nakorn Ratchasima Hospital in Nakorn Ratchasima, and Srinagarind Hospital in Khon Kaen. Exclusion criteria included history of prematurity (< 34 weeks of gestational age), birth weight < 1500 g, multiple illnesses at birth, or other additional genetic disorders. Both GH and non-GH received patients were included, but the data after receiving GH were excluded before curve construction. Longitudinal data for weight, length/height, and head circumference were collected from medical records and national mother and child health handbooks with aged between 0 and 18 years. Head circumferences (HC) measurements were gathered between 0 and 6 years. Baseline demographic data, including gender, current age, age at diagnosis, initial presentations, comorbidities, and GH treatment were reviewed.

Generalized Least Squares (GLS) lead to better goodness-of-fit indices compared to LMS method when the number of data are small [14], therefore, growth charts in this study were constructed using the GLS methods. Curve smoothing was performed by fractional polynomials and exponential transformation [15]. All procedures were done with statistical software STATA v.15.1 (StataCorp. 2017. College Station, TX: StataCorp LLC). Separated height and weight curves in each gender were constructed representing 7 centile ranges (3^rd^, 10^th^, 25^th^, 50^th^, 75^th^, 90^th^, 97^th^) for the ages of 0–36 months and 3–18 years. HC in each gender was presented in 7 centiles between 0 to 6 years. Growth charts for weight, height and head circumference for Thai PWS compared to the 50^th^ normative centile from the Thai reference growth data (Ministry of Public Health, 1999) were constructed.

## Results

### Clinical data

One hundred and thirty-five children with PWS were initially reviewed (97 from Ramathibodi Hospital, 23 from Srinagarind Hospital, 8 from Maharat Nakorn Ratchasima Hospital and 7 from the Thai PWS support group). Twenty-two cases were excluded due to prematurity, birth weight less than 1500 g (13 patients) and/or unavailable growth parameters (9 patients). Thus, a total of 113 Thai patients (55 males and 58 females) were finally enrolled. Mean age of diagnosis was 28.8 months (0–238 months). Fifty percent of patients were diagnosed less than 6 months of age. The early diagnosed patients mostly presented with poor feeding and hypotonia. Uncommon presentations, such as sepsis-like illnesses, were noted in four patients. Patients, who were diagnosed after 2 years of age, mostly presented with obesity, snoring, and developmental delay. Fifty percent of patients were diagnosed by the FISH study and the remainder by *SNRPN* methylation specific PCR. Twenty percent of patients confirmed by methylation test initially had negative FISH study.

Three patients had significant pharyngeal incoordination, requiring gastrostomy tube at 6–7 months of age, and can be orally fed within 2–3 years of age. Sixty-five percent (36/55) of male PWS were diagnosed with undescended testis/testes. Hypothyroidism was noted in 13 patients. Fifteen patients had skeletal abnormalities such as genu valgus or scoliosis in late childhood. Obesity was the most common comorbidity with mean age of diagnosis of 5 years. Other manifestations included obstructive sleep apnea (44), hypertension (16), diabetes mellitus (31), and dyslipidemia (22). Secondary sex characteristics were noted in 38% (26/68) of the patients older than 8 years. Fourteen patients (12%) had received GH therapy, and their growth parameter data after the GH treatment were excluded.

### Growth curves

**Fig. 1:**
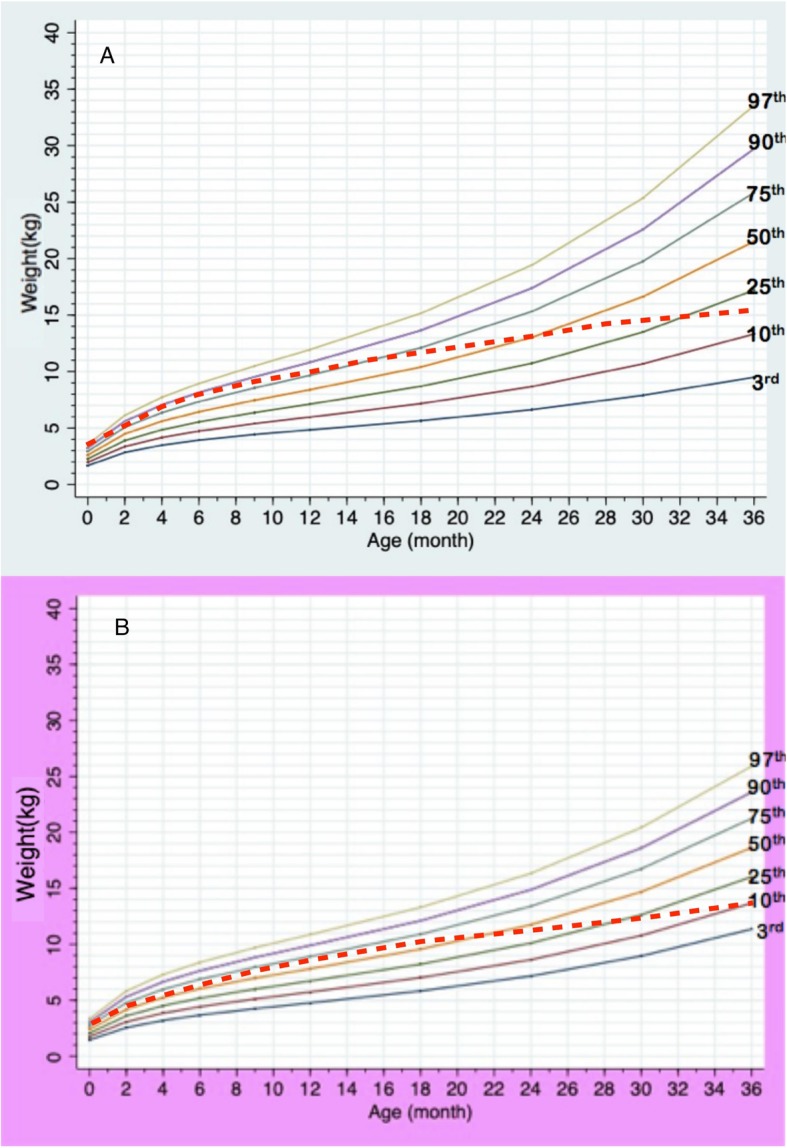
Growth charts for weight at age of 0–36 months in Thai PWS males (**a**) and females (**b**) compared with the 50^th^ normative centile (dashed line)

**Fig. 2:**
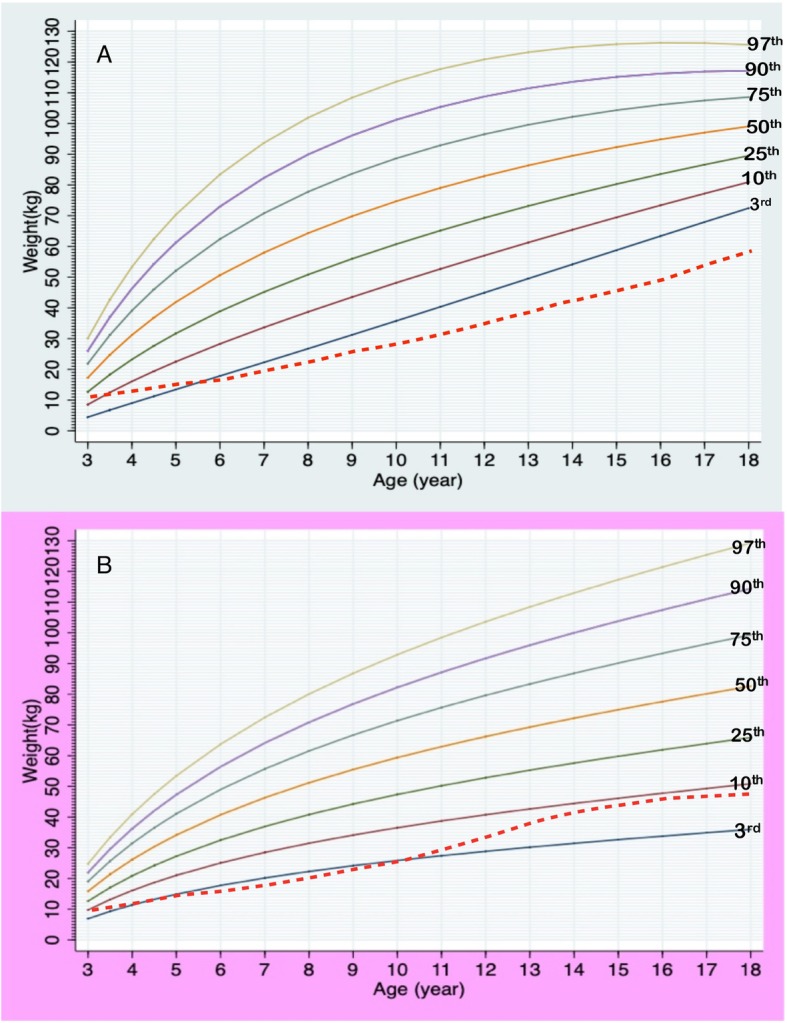
Growth charts for weight at ages of 3–18 years old in Thai PWS males (**a**) and females (**b**) compared with the 50^th^ normative centile (dashed line)

**Fig. 3:**
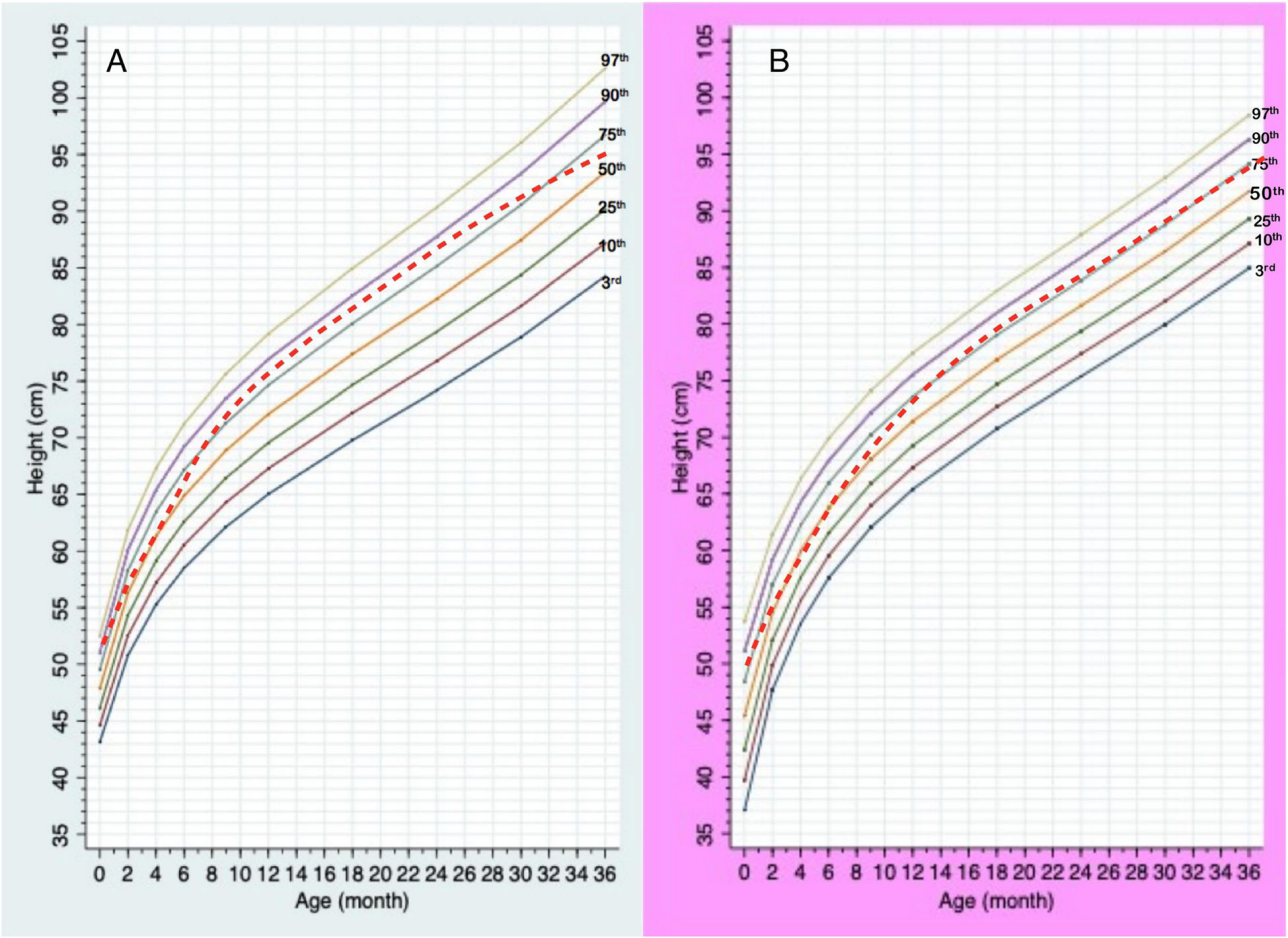
Growth charts for length/height at ages of 0–36 months in Thai PWS males (**a**) and females (**b**) compared with the 50^th^ normative centile (dashed line)

**Fig. 4:**
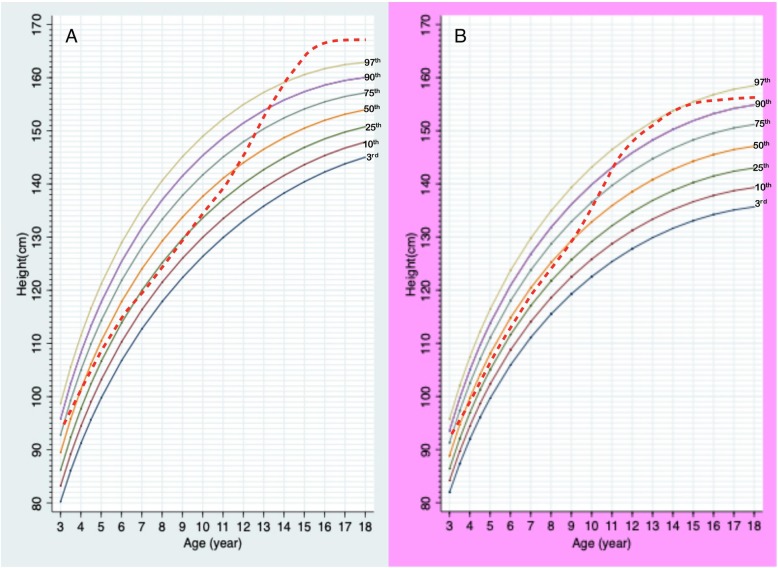
Growth charts for height at ages of 3–18 years old in Thai PWS males (**a**) and females (**b**) compared with 50^th^ centile of normal population (dashed line)

**Fig. 5:**
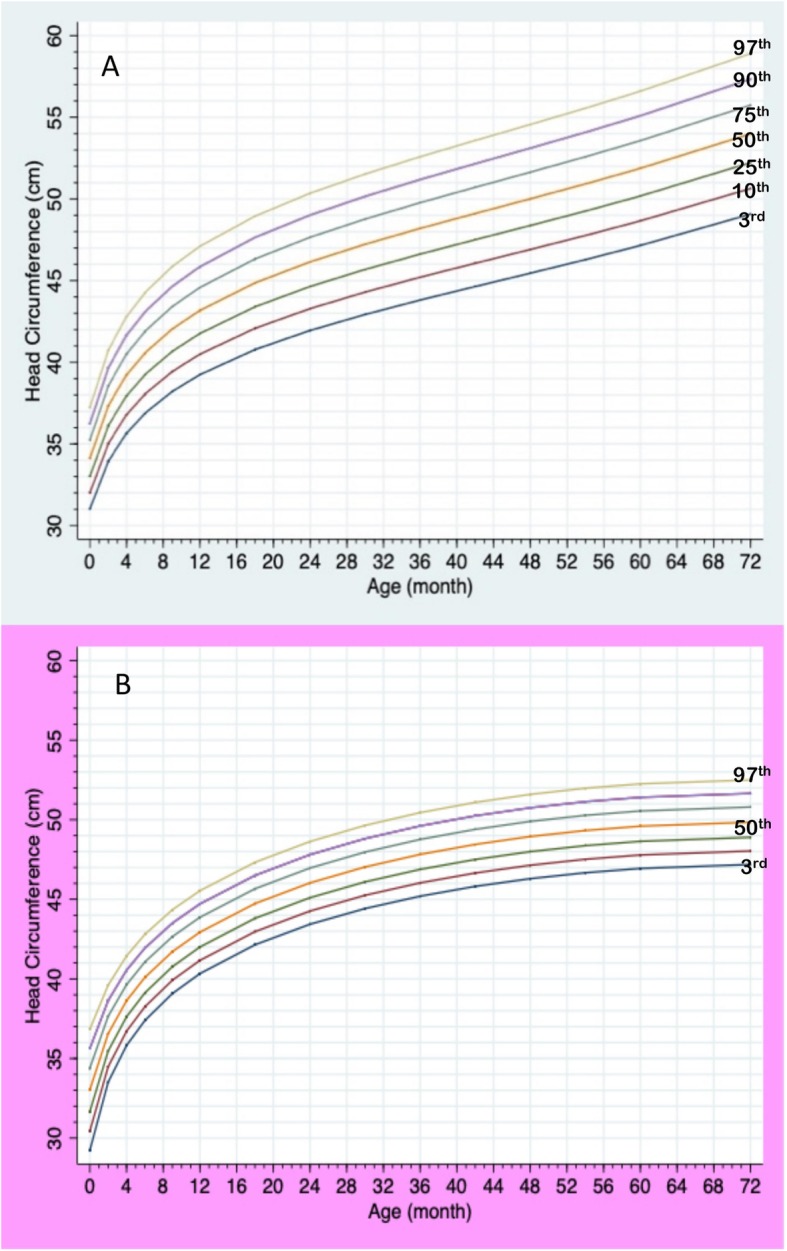
Growth charts for head circumference at ages of 0–6 years old in Thai PWS males (**a**) and females (**b**)

Total data points included 896, 765, and 283 points for weight, height, and HC, respectively. Information of data points in each gender and age group is presented in Table 1. Median numbers of measurements per patient were 4 in a boy aged 0–3 years (interquartile range or IQR 2–7), 4 in a boy aged 3–18 years (IQR 2–5), 6 in a girl aged 0–3 years (IQR 2–8), and 4 in a girl aged 3–18 years (IQR 2–9). Mean birth weight was 2.6 kg (2.7 kg in males; 2.5 kg in females), and mean birth length was 48 cm (49 cm in males; 48.7 cm in females). The growth charts of Thai PWS compared to the 50^th^ normative centile from the Thai national growth reference are shown in Figs. 1, 2, 3, 4 and 5. For weight, the 50^th^ centile of Thai PWS was less than the 50^th^ normative centile until 2 years of age in both genders (Fig. 1). After 2 years of age, rate of weight gain significantly increased in both genders. After 4 years of age, the 3^rd^ centiles are above the 50^th^ centile of typical Thai children curves in both genders (Fig. 2).

**Table 1:** Data points in all growth parameters

Age (years)	Weight (points)		Length/height (points)		HC (points)	
	Male	Female	Male	Female	Male	Female
0–1	163 (41%)	181 (37%)	129 (38%)	136 (31%)	93 (75%)	89 (56%)
1–3	81 (20%)	93 (19%)	67 (20%)	89 (20%)	19 (15%)	37 (24%)
3–6	73 (18%)	95 (19%)	67 (20%)	92 (21%)	13 (10%)	32 (20%)
7–12	46 (12%)	85 (17%)	44 (13%)	85 (19%)	–	–
> 12	37 (9%)	42 (8%)	32 (9%)	42 (9%)	–	–
Total data points	400	496	339	426	125	158

*HC* head circumference

For height, the 50^th^ centile in PWS was lower than the normative 50^th^ centile in all ages and genders (Figs. 3 and 4). The height curve showed no growth spurt in both genders (Fig. 4). Final height at 18 years of age was approximately 14 cm and 10 cm below the average normative height in males and females, respectively (Fig. 4). Head circumference curves in males showed a wide distribution (Fig. 5).

## Discussion

This study, the first in Southeast Asian population, developed growth charts for Thai non-GH treated children with PWS. The curves will be helpful for monitoring and prediction of growth velocity in Thai patients with PWS. The ideal outcome for children with PWS after dietary intervention and growth hormone therapy is having normal growth. In many countries, such as Thailand, most children with PWS have not received GH treatment. Therefore, the disease-specific growth chart will help pediatric nutritionists and geneticists to accurately predict weight and height, and closely monitor them to initiate early intervention. These charts will be useful for the comparison of growth outcome between the PWS children with and without GH treatment in our population.

LMS method is widely used to generate growth chart. Based on a previous study, GLS method lead to better goodness-of-fit indices compared to LMS method when the number of small [14]. We generated the growth curves by using GLS method instead of LMS method because the number of data in the study were quite small. Previous reports constructed specific growth curves for PWS in the US, Germany, Korea, and Japan [5–7, 9, 16]. Study of growth patterns in China has been reported without growth curve construction [17]. The growth pattern of Thai PWS individuals was similar to the previous literature from several countries [5–7, 9, 16]. The mean birth weight in Thai PWS (2.7 kg, male; 2.5 kg, female) was below the normal newborn (3.4 kg, males; 3.2 kg, females), and was quite similar to the studies in Japan (2.7 kg, males; 2.62 kg, females), Korea (2.67 kg, males; 2.79 kg, females), and China (2.8 kg, males; 2.77 kg, females) [5, 6, 17]. The mean body weight was below the average values for typical children before 2 years of age, then weight velocity definitely increased after 2 years of age in both genders. For the height curves of 0–36 months, the 50^th^ centile of PWS curve was below the 50^th^ centile of the typical children’s curve. The mean height was close to normal children between aged of 3–10 years, then the height after 10 years of age is obviously lower than normal children because of no growth spurt. This finding was observed in other studies [5, 9, 16].

Previous literature demonstrated growth differences among individuals of different ethnicities [18]. The height and weight of American and African-American seems to be taller and bigger than the Korean and Japanese children with PWS [5, 6, 16, 19]. Interestingly, the average weight of Thai PWS at 18 years of age in our study was slightly heavier in males, 99 vs 94 kg, but lower in females, 82 vs 86 kg, as compared to the US affected population [19]. We cannot explain the reason underlying this discrepancy; however, it could be due to better environmental/dietary control for the girls over the boys in Thai affected population, recruitment bias, different socioeconomic backgrounds such as the levels of parental education and exercise habit.

The mean height at 18 years of age of Thai PWS is lower than that of the US (154 vs 160 cm in males; 146 vs 147 cm in females) and higher than those reported in Japanese patients (154 vs 150 cm in males; 146 vs 141 cm in females) [5, 16]. The direction of differences is similar to the differences between unaffected growth charts in Thai and the US (170 vs 176 cm in males; 158 vs 163 cm in females), based on the Thai Reference Growth Data, Ministry of Health 1999 and the growth data from Center for Disease Control (https://www.cdc.gov/growthcharts/charts.htm). However, the mean heights at 18 years of age in unaffected Thai and Japanese are alike (170 in males and 158 in females) [20, 21]. Interestingly, our study showed that the mean height of Thai PWS was higher than those of Japanese PWS. The reason for this difference is unknown; however, possible explanation include confounding variables such as the difference of parental heights of the patients enrolled in the present and the Japanese study which could affect the final heights of PWS individual [5]. Unfortunately, the data of parental heights from both studies were not available for further investigation to support this proposition.

Over 50% of children with PWS are growth hormone (GH) deficient by standard testing protocols [10, 22, 23]. Subnormal GH response during hormone provocation tests do not definitely indicate pathological GH deficiency. Benefits from GH therapy from infancy through adulthood have been demonstrated including improvement in weight control, cognitive function, tone, and head size in multiple reports [10, 11, 24, 25]. An expert consensus guideline for GH treatment in PWS has been published [10]. However, most of Thai children with PWS have not received GH due to lack of reimbursement of GH under the national universal coverage. In this study, only 14 patients (12%) received GH treatment, and the data after GH treatment were not included to construct the curves. The median age of GH initiation was 2 years and 9 months (ranging from 1 month to 5 years and 5 months). Prior to the GH treatment, these patients did not have major differences compared with non-GH treatment patients including growth or health problems except one patient presenting with neonatal persistent hypoglycemia and one patient required noninvasive respiratory support due to hypoventilation in neonatal period. The results of clonidine and levodopa stimulation test were available in eleven patients, which showed low or subnormal responses. However, the scatter plots of their growth parameters before GH treatment were in 25^th^ - 75^th^ centile of Thai PWS growth charts. This data supports that the fourteen patients before GH treatment were not have severe growth failure. The scatter plots of weight data after GH treatment from these 14 patients aged 3–18 years were not in excess of the 50^th^ centile in the Thai PWS growth charts. This finding represents that weight control in GH-treated patients is better than non-GH treated patients. Height in these GH-treated patients distributed in all centiles. In the future, this information may be helpful to convince policy makers to support coverage of GH therapy in Thailand.

This study was performed in multiple centers from different areas in Thailand and can represent the data of the Thai PWS population. However, this study has some limitations due to limited numbers of patients especially the HC parameter. The technique of measurement cannot be validated since this is a retrospective design. However, the anthropometric parameters of children are normally measured by well-trained health professionals. This was the best resource for this study. Another limitation is that various factors related to growth pattern, including comorbidities and nutritional intervention and management, were not controlled for in our analysis. The correlation study about factors related to the growth pattern in PWS would be interesting in further research.

## Conclusion

This is the first study to construct specific growth curves for PWS in Thailand. The curves can be used in clinical application and growth monitoring for Thai and other Southeast Asian population. Furthermore, they could be used to demonstrate response of GH therapy compared to non-GH therapy in PWS, which ideally can encourage GH treatment in Thailand in the future. These data in Thai children with PWS also illustrate the importance of growth curve development in diverse populations.

## Abbreviation

GH: growth hormone
GLS: Generalized Least Squares
HC: head circumference
PWS: Prader-Willi syndrome
